# Analysis of antioxidant effect of two tripeptides isolated from fermented grains (Jiupei) and the antioxidative interaction with 4‐methylguaiacol, 4‐ethylguaiacol, and vanillin

**DOI:** 10.1002/fsn3.1100

**Published:** 2019-06-14

**Authors:** Yunsong Jiang, Dongrui Zhao, Jinyuan Sun, Xuelian Luo, Hehe Li, Xiaotao Sun, Fuping Zheng

**Affiliations:** ^1^ Beijing Advanced Innovation Center for Food Nutrition and Human Health Beijing Technology & Business University (BTBU) Beijing China; ^2^ Beijing Laboratory for Food Quality and Safety Beijing Technology & Business University (BTBU) Beijing China; ^3^ State Key Laboratory of Infectious Disease Prevention and Control, National Insititue for Communicable Disease Control and Prevention, Collaborative Innovation Centre for Diagnosis and Treatment of Infectious Disease Chinese Centre for Disease Control and Prevention Beijing China

**Keywords:** antioxidant tripeptides, HepG2 cells, interaction effect, Jiupei, phenols

## Abstract

Jiupei (fermented grains) is the raw material for Baijiu distillation. The antioxidant activities of peptides Val‐Asn‐Pro (VNP) and Tyr‐Gly‐Asp (YGD) identified from Jiupei were evaluated according to in vitro chemical assays (e.g., 2,2′‐azino‐bis (3‐ethylbenzothiazoline‐6‐sulfonic acid) diammonium salt, 1,1‐diphenyl‐2‐picrylhydrazyl, and oxygen radical absorbance capacity) and 2,2′‐azobis (2‐methylpropionamide)‐dihydrochloride‐activated HepG2 cell model. The interaction on antioxidant activities between peptides (VNP and YGD) and three functional phenols (4‐methylguaiacol, 4‐ethylguaiacol, and vanillin) which were found in Baijiu was also measured. On the basis of the results, two peptides exhibited strong antioxidant ability in oxygen radical absorbance capacity (ORAC) assay. Furthermore, they suppressed the generation of reactive oxygen species. The intracellular antioxidant enzymatic system and nonenzymatic system were also regulated by VNP and YGD. In addition, it was confirmed that partial auxo‐action between peptides and phenols appeared mostly in chemical assays. The findings above might indicate that VNP and YGD are potent natural antioxidants in Jiupei even in Baijiu through distillation process and lay the foundation for illustrating the interactions among different functional substances in Baijiu.

## 1 INTRODUCTION

Oxidative stress (OS) refers to the imbalance between oxidation and antioxidation in vivo. It is a negative effect produced by free radicals in the body and is considered to be an important factor leading to aging and diseases (Zhao et al., 2017). OS is mainly caused by reactive oxygen species (ROS), which is a by‐product of biological aerobic metabolism, including oxygen ions, peroxides, and oxygen‐free radicals (Caro et al., 2018). Appropriate amount of ROS can act as a signal molecule of signal transduction and play an important role in the development, growth, differentiation, and regeneration of cell organism (Wójciak et al., 2018; Zhao, Cheng, et al., 2018). However, the rapid increase in ROS can change the structure of some protein in the human body; thus, cell differentiation, regeneration, apoptosis, and damage of cell structures can be affected (Joy et al., 2017). Given the diversity of the ways that ROS is produced, negative impact (e.g., aging and death) on the cells and tissues in the body will happen when ROS content beyond the normal range (Piotr, Mateusz, & Danuta, 2018). In addition to the effects of ROS, other factors such as inflammation, cardiovascular, psychic factor, environment, and unhealthy lifestyles can also cause oxidative stress (OS) (Islam, Kabir, Inoue, Sada, & Kakugo, 2016; Korsager & Matchkov, 2016; Omidi, Vakili, Nazifi, & Parker, 2016). Therefore, finding efficacious ways to scavenge superfluous‐free radicals and to maintain them at appropriate levels is a quite practical hot issue.

Animals and plants can be selected as the appropriate subjects to extract natural antioxidants which possess high nutritional values (Bordbar, Ebrahimpour, Zarei, Abdul Hamid, & Saari, 2018; Cömert & Gökmen, 2018; Mahdavi‐Yekta, Nouri, & Azizi, 2019). These natural antioxidants can protect the body from excessive OS. Peptide is regarded as an ideal natural antioxidant substance. Some studies (Feng, Ruan, Jin, Xu, & Wang, 2018; Gallego, Mora, & Toldrá, 2018; Suwal, Ketnawa, Liceaga, & Huang, 2018) have focused on antioxidant peptides derived from various kinds of food, peptide AEEEYPDL was found with antioxidant activity in dry‐cured ham, and five novel peptides (VYTE, TKGQ, MMLQK, TPAIS, and VSAFLA) isolated from Chinese chestnut protein also showed bioactivity and rainbow trout contains functional peptides as well. Peptides found in foods are safe for humans and potentially useful for human health in virtue of their low toxicity, high activity, and easy absorptivity (Fang, Xu, Lin, Cai, & Wang, 2019; Lin et al., 2013). Antioxidant peptides are among the most studied bioactive peptides, and the antioxidant capacity of a peptide is usually related to its molecular weight, specific amino acid sequence, and space structure (Ghribi et al., 2015). These results suggest that antioxidant peptides isolated from animals and food can be used as food additives to enhance the healthy value of foodstuffs even in pharmaceutical field.

Nowadays, a variety of antioxidant peptides have been identified from meat muscles, animals, vegetables, fruits, plant protein, and other foods (Gao et al., 2019; Wu et al., 2018; Yuan et al., 2018; Zhang & Mu, 2017; Zhang, Gao, et al., 2018; Zhao, Luo, et al., 2018; Zhu, Zhang, Zhou, & Xu, 2016). Peptide GWWW isolated from myoglobin showed a strong antioxidant activity against peroxyl radicals, and they suggested that amino acid side chains can be a base to design peptides with activities against some special target ROS and RNS (Karadag, Ozcelik, & Saner, 2009). The antioxidant abilities of peptides were demonstrated both in vitro and in vivo (Wang et al., 2016; Zeng, Sun, Zhang, Liao, & Shi, 2017). For example, two peptides VYTE and VSAFLA isolated from Chinese chestnut protein were suggested to exert a high ,2′‐azino‐bis (3‐ethylbenzothiazoline‐6‐sulfonic acid) diammonium salt (ABTS) scavenging capacity (Feng et al., 2018). Additionally, a peptide CERPTCCEHS was found possess a strong capability to enhance the activities of antioxidant enzyme, including superoxide dismutase (SOD), catalase (CAT), and glutathione peroxidase (GSH‐Px). It also led to an important decrease in malondialdehyde (MDA) level in animal serums (Zeng et al., 2017).

**Figure 1:**
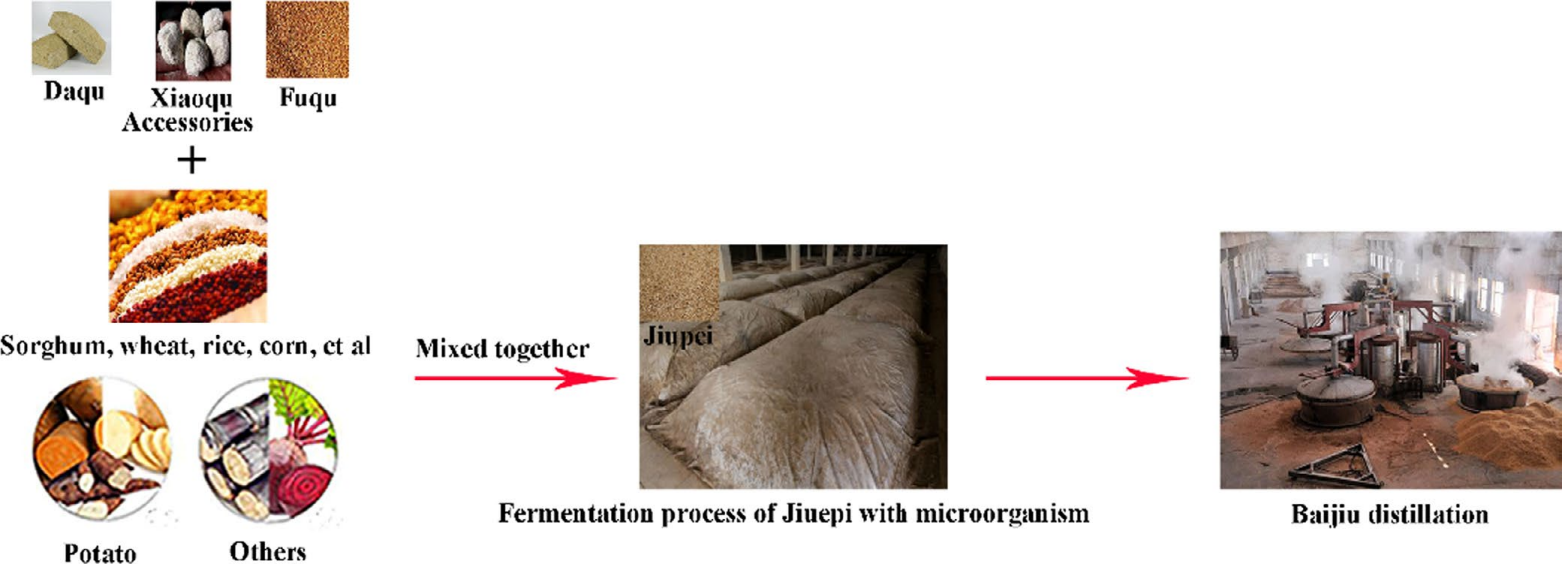
Introduction of Jiupei (raw material of Baijiu distillation) and Baijiu distillation process

Chinese Baijiu is a traditional indigenous distilled spirit with a long history, unique technology, various varieties, and relatively large output. It has been loved by the broad masses of people. Baijiu is produced by a solid‐state fermentation along with a solid‐state distillation process (Liu & Sun, 2018). Studies have reported that moderate consumption of Baijiu benefits human health based on the various healthy volatile and nonvolatile trace components in it (Zheng & Han, 2016). For instance, phenols are kinds of antioxidants in foods and beverages, which exhibited beneficial effects on human health (Rao et al., 2018). Phenols such as 4‐methylguaiacol (4‐MG), 4‐ethylguaiacol (4‐EG), and vanillin (VA) are important aromatic compounds already found in Baijiu, which also present antioxidant activities both in vitro and in vivo (Zhao et al., 2017). A lot of volatile components have been reported as the functional substances in Baijiu; however, more and more attention has also been paid to the nonvolatile components such as peptides in Baijiu (Wu et al., 2017) now. Jiupei is used as the raw material of Baijiu distillation (Figure 1). Since grains possess relatively high content of protein, during the fermentation process, these proteins are decomposed into peptides by microorganisms (Liu & Sun, 2018). Therefore, Jiupei can be used as a good object for functional peptide extraction and a basis of tracing peptides identified in Baijiu. Potential interaction between peptides that may be distilled into Baijiu and other functional substances already proved exist in Baijiu is worthy of study considering the healthy impact they will bring into Baijiu.

In this study, the main purpose was to measure the identified peptides’ (Zhang, Jiang, et al., 2018) antioxidant activities with ABTS, 1,1‐diphenyl‐2‐picrylhydrazyl (DPPH), ORAC, reducing power (RP), and ferrous chelating ability (FCA) assays. The effect of protection against 2,2′‐azobis (2‐methylpropionamide)‐dihydrochloride (AAPH)‐induced oxidative damage in human hepatoma cells (HepG2 cells) was also investigated by measuring the levels of OS biomarkers [MDA, oxidized glutathione (GSSG), and glutathione (GSH)] and antioxidant enzymes (SOD, CAT, and GSH‐Px). The antioxidant interaction between peptides [Val‐Asn‐Pro (VNP), Tyr‐Gly‐Asp, (YGD)] and three phenols (4‐EG, 4‐MG, and VA) was also initially measured through in vitro assays so as to provide a reference for the study of interaction among different kinds of functional substances in Baijiu.

## 2 MATERIALS AND METHODS

### 2.1 Materials and reagents

Jiupei of Guojing sesame flavor‐type Baijiu was kindly provided by Bandaojing Co., Ltd. 6‐hydroxy‐2,5,7,8‐tetramethylchroman‐2‐carboxylic acid (Trolox) and ABTS kit were obtained from Beyotime Biotechnology Co., Ltd. Trichloroacetic acid (TCA) was purchased from Tianjin Guangfu Fine Chemical Industry Research Institute. DPPH was purchased from Sigma‐Aldrich. AAPH was obtained from Yuanye Bio‐Technology Co., Ltd. Potassium ferricyanide and ferrous chloride (purity >98%) were obtained from Xilong Scientific. Iron chloride (purity >98%) was purchased from J&K Scientific Co., Ltd. Ferrozine was obtained from Solarbio Life Sciences Co., Ltd. Ethylenediaminetetraacetic acid disodium salt was purchased from Biotopped Technology Co., Ltd. PBS phosphate buffer (pH 7.4, 50, 75, 100 μM) was obtained from Yuanye Bio‐Technology Co., Ltd. PBS phosphate buffer (pH 6.5, 0.01 μM) was obtained from Bayer Biotechnology Co., Ltd. Fluorescein disodium (FL) was obtained from TCI Development Co., Ltd. Absolute ethanol (purity >99.7%) was obtained from Sinopharm Chemical Reagent Co., Ltd. All the chemicals and reagents used were of analytical grade.

Val‐Asn‐Pro and YGD (purity >98%) were purchased from Shanghai Gil Biological Science and Technology Co., Ltd. Microscale MDA, SOD, CAT, GSH‐Px, and GSH/GSSG assay kits were purchased from Nanjing Jiancheng Institute of Biotechnology. Bicinchoninic acid (BCA) protein assay kit was purchased from Beijing Solarbio Science & Technology Co., Ltd. Cell counting kit (CCK‐8) was purchased from Bimake. Other reagents and instruments were described in the specific experimental steps.

### 2.2 The extraction yields of VNP and YGD

**Figure 2:**
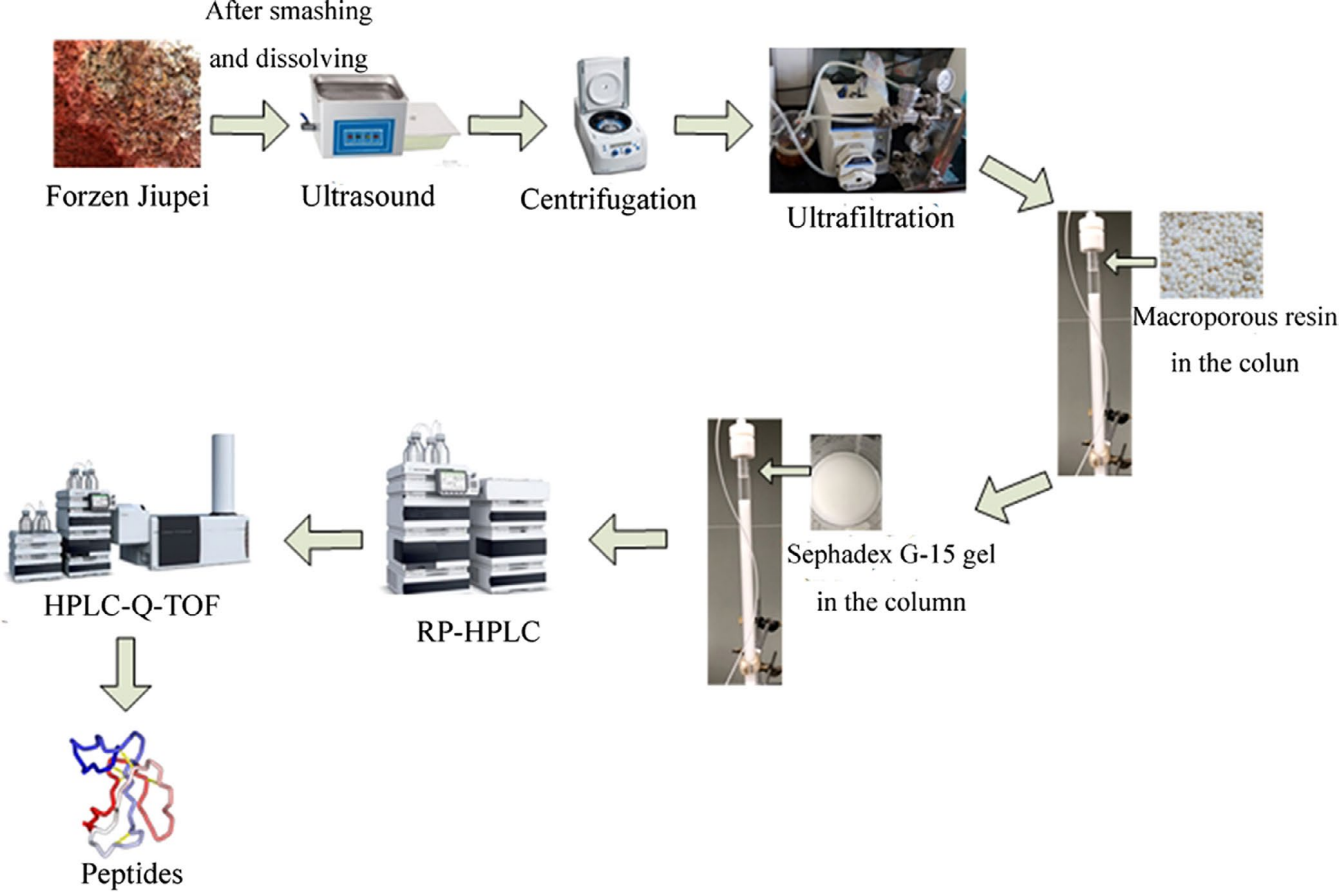
Isolation, purification, and identification processes of peptides

Fifty milliliter peptides’ extract solution was obtained from 400 g frozen Jiupei. The identification process of two peptides was summarized in Figure 2 according to our previous study (Zhang, Jiang, et al., 2018). The extraction yield of two peptides in solution was measured by high‐performance liquid chromatography (HPLC) with external standard method (Liu et al., 2015). The parameters and elution program were the same as Zhang's method (Zhang, Jiang, et al., 2018). The synthetics of VNP and YGD were used as reference standards.

### 2.3 In vitro antioxidant experiments of VNP and YGD

#### 2.3.1 ABTS assay

ABTS assay was conducted based on a previous method with some modifications (Zheng, Zhao, Dong, Su, & Zhao, 2016). Briefly, 150 μl ABTS^+^ radical solution was mixed with 50 μl working standard solution of VNP, YGD, or 50 μl ultrapure water as the blank control. After incubation for 30 min at room temperature, the absorbance at 734 nm was measured with a microplate reader (Molecular Devices). The antioxidant capacities of VNP and YGD were determined as a percentage decrease in color intensity (% of inhibition) related to the blank control sample (Equation (1)). Trolox was regarded as the standard antioxidant, and the results were expressed as μmol Trolox equivalent (TE)/μmol sample.

Twenty milligram per litter 4‐EG, 4‐MG, and VA were selected according to Zhao's assays (Zhao et al., 2017) to mix with 4,000, 2,000, 1,000, 500, and 250 mg/L YGD (VNP exhibited little ABTS radical scavenging ability) during the interactive assays. The mixture (25 μl for both peptide and phenols) was used as working solution. The method of ABTS assays was the same as the method above.

(1) $$\text{inhibition(}\%\text{)}=\frac{\text{Ablank}\;\text{control}-\text{Asample}}{\text{Ablank}\;\text{control}}\times 100\%.$$

#### 2.3.2 DPPH assay

DPPH radical scavenging activities of peptides were assessed as Mareček's et al., 2017) method described, with some modifications. In brief, 100 μl DPPH ethanolic solution was added to 100 μl working standard solution of VNP and YGD or 100 μl ultrapure water (Millipore) as the blank. All the reactions were left in the dark at room temperature for 40 min. Afterward, the absorbance was measured at 517 nm using the microplate reader. The antioxidant capacities of two peptides were determined as a percentage decrease in color intensity (% of inhibition) related to the blank control sample (Equation (2)). Total antioxidant capacity of each peptide was expressed as an equivalent of Trolox (TEAC) per 1 g of dry matter of a sample (μmol/g).

Fifty milligram per litter 4‐EG and 4‐MG were selected according to Zhao's assays (Zhao et al., 2017) to mix with 4,000, 2,000, 1,000, 500, and 250 mg/L VNP (YGD showed no DPPH radical scavenging ability) during the interactive assays. The mixture of 50 μl peptides and 50 μl phenols was used as working solution. The method of DPPH assays was the same as the method above.

(2) $$\text{inhibition(}\%\text{)}=\left(1-\frac{\text{Asample}-\text{Acontrol}}{\text{Ablank}\;\text{blank}}\right)\times 100\%.$$

#### 2.3.3 ORAC assay

The modified ORAC assay was experimented according to the method described by Garrett et al. (2014). One hundred twenty microliter FL solution (120 nM) was mixed with each sample standard solution (VNP and YGD) in a well of a black 96‐well microplate. Twenty microliter ultrapure water was used as the blank control. The reaction was conducted at 37°C for 15 min. After that, 60 μl AAPH (40mM) was added to the black 96‐well microplate using a multichannel pipette (Eppendorf, Hamburg, Germany) and shaken for 30 s. Jie‐Kun, Yao, and Kurihara (2006) method was used to calculate the ORAC values. The final results were expressed as μmol TE/μmol peptide.

#### 2.3.4 RP assay

Reducing power assay was based on a previous Zheng's method (Zheng, Zhao, Dong, et al., 2016) with appropriate modifications. Briefly, 0.5 ml peptide sample, PBS (200 mM, pH 6.6), and 1% (w/v) potassium ferricyanide were mixed together in the centrifuge tube and incubated for 20 min at 50°C. Then, 0.5 ml 10% (w/v) TCA, 2 ml ultrapure water, and 0.4 ml 1% (w/v) FeCl_3_ were added to the mixture and placed at room temperature for 10 min. After that, the adsorption at 700 nm was measured by a microplate reader. The PBS (pH 6.6) was used instead of the peptides as the blank control. The results were expressed as μmol TE/μmol peptide.

One hundred milligram per litter 4‐EG, 4‐MG, and VA were selected according to Zhao's assays (Zhao et al., 2017) to mix with 4,000, 2,000, 1,000, 500, and 250 mg/L YGD during the interactive assays. The mixture of peptides and phenols (both 0.25 ml) was used as working solution. The method of RP assays was consistent with the method above.

#### 2.3.5 FCA assay

Ferrous chelating ability assay was determined by the method of Zheng, Zhao, Dong, et al. (2016). The ultrapure water was used instead of the peptides as the blank control. Ethylenediaminetetraacetic acid (EDTA) was used as the standard antioxidant, and the results were expressed as μmol EDTA equivalent (EE)/μmol peptide.

Three phenols showed no FCA, so there was no interactive assay between peptides.

### 2.4 In vivo antioxidant experiments of VNP and YGD

#### 2.4.1 HepG2 cell culture

Human hepatic cell line HepG2 (Wu et al., 2018; Zhao et al., 2017) was kindly provided by National Institute for Communicable Disease Control and Prevention, Chinese Centre for Disease Control and Prevention. Cells were grown and maintained in a humidified incubator at 37°C and 5% CO_2_ using Dulbecco's modified Eagle's Medium (Mediatech) provided with 10% (v/v) fetal bovine serum obtained from Gibco. The culture medium was changed every 2 days and was split at 80%–90% confluency using 0.25% trypsin and 0.02% EDTA.

#### 2.4.2 Measurement of HepG2 cell viability treated by YGD and VNP

The HepG2 cells (1 × 10^5^/ml) growing well in 96‐well microplate were treated with different concentrations of peptides. The cell viability was measured according to the cell counting kit (CCK‐8 assay) (Su et al., 2017). Ten microliter of CCK‐8 was added into the 96‐well microplate with cells. The same volume of serum‐free medium was added as the control group. The absorbance of each well was measured at 450 nm using a microplate reader. Cell viabilities were calculated based on the percentage of the absorbance compared with control group.

#### 2.4.3 Assays of peptides’ effect on ROS of HepG2 cells induced by AAPH

The HepG2 cells (1 × 10^5^/ml) in 96‐well microplate were grown under a condition of 37°C and 5% CO_2_ for 24 hr. Afterward, the medium was removed and different concentrations of peptides in 100 μl serum‐free medium containing 10 μmol/L of DCFH‐DA were added into cells and cultured for 60 min. After that, the medium was removed and the cells were washed with serum‐free medium for three times. One hundred microliter serum‐free medium containing 200 μM AAPH was then added into the cells. After incubation of 0, 30, 60, 90, 120, and 180 min, the fluorescence of the sample was measured using microplate reader under excitation wavelength‐488 nm and emission wavelength‐525 nm. Cells treated with AAPH alone were used as AAPH group. Cells only treated with DCFH‐DA and mixed with 100 μl of serum‐free medium were regarded as the blank control group.

#### 2.4.4 Determination of peptides’ activity on BCA, GSH, GSSG, and MDA contents

The HepG2 cells (1 × 10^5^/ml) in 24‐well microplate were grown with a condition of 37°C and 5% CO_2_ for 24 hr; then, the medium was removed and the cells were washed by PBS buffer. After that, serum‐free medium containing 200 μM AAPH was added into cells. Cells treated with peptides were denoted as sample groups, and untreated cells were used as control group. Afterward, the medium was removed, and the cells were washed by PBS for three times. The cells were then digested by RIPA buffer with 1 mΜ PFSM for 10 min and centrifuged at 14,000 *g* for 5 min to obtain the supernatant. The contents of BCA, GSH, GSSG, and MDA were measured according to the illustrations from the assay kits. The results were finally calculated by dividing the normalized data of each group by those in the control group.

#### 2.4.5 Determination of peptides’ activity on SOD, CAT, and GSH‐Px

Cell protein supernatant was obtained with the same method above, and the activities of SOD, CAT, and GSH‐Px were measured by the assay kits. The results were expressed as relative value contrasted to control group.

### 2.5 Statistical analysis

Experiments were performed at least in triplicate. The data obtained from all the experiments were represented as the mean ± standard deviation (*SD*). Statistical differences among groups were obtained using the one‐way ANOVA test with SPSS (version 19.0, IBM Inc.). *p* < 0.05 was accepted as statistically significant.

## 3 RESULTS AND DISCUSSION

### 3.1 The extraction yields of VNP and YGD

**Figure 3:**
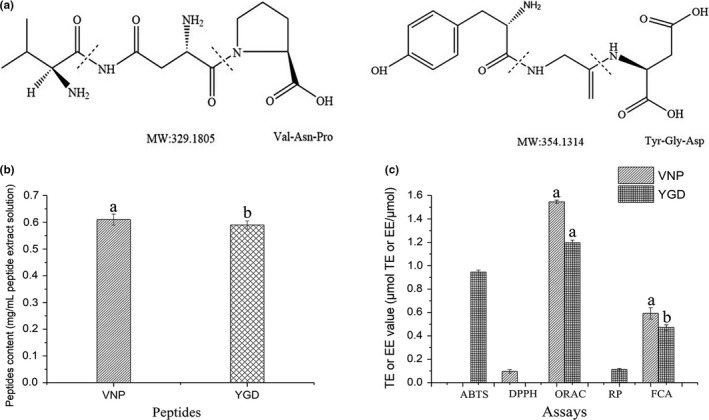
The chemical structures of tripeptides Val‐Asn‐Pro (VNP) and Tyr‐Gly‐Asp (YGD) (a). The extraction contents of VNP and YGD in Jiupei extract solution (b). Antioxidant activities of VNP and YGD determined in ,2′‐azino‐bis (3‐ethylbenzothiazoline‐6‐sulfonic acid) diammonium salt, 1,1‐diphenyl‐2‐picrylhydrazyl, oxygen radical absorbance capacity, reducing power, and ferrous chelating ability assays (c)

The structures of VNP and YGD were presented in Figure 3a. The extraction yields of two tripeptides were measured by HPLC and showed in Figure 3b. The contents of two tripeptides are 0.61 mg/ml peptide extract solution for VNP and 0.59 mg/ml peptide extract solution for YGD, respectively.

The existence of peptides in Jiupei provided the evidence for originating the source of peptides in Baijiu (Wu et al., 2017), and the content of functional peptides in Baijiu can be increased by optimizing the distillation conditions in the future. It is also an ideal selection when isolating functional peptides from Jiuzao (residue after Baijiu distillation) considering a content of protein and peptides left in it after distillation.

### 3.2 In vitro antioxidant activities of VNP and YGD measured by ABTS, DPPH, ORAC, RP, and FCA assays

Figure 3c exhibited the activities of two peptides in antioxidant chemical‐based assays. VNP exhibited a strong activity in ORAC assay following a value of 1.55 μmol TE μmol VNP. It also showed an activity of FCA with a value of 0.59 μmol EE/μmol VNP and a DPPH radical scavenging activity with a value of 0.10 μmol TE/μmol VNP. YGD showed its abilities in four assays; in ORAC and ABTS assays, it exhibited strong antioxidant activities. It had a strongest absorbance activity with a TE value of 1.20 μmol TE/μmol YGD in ORAC assay, following the ABTS radical scavenging activity, with a TE value of 0.95 μmol TE/μmol YGD. In other two assays, YGD showed a TE value of 0.11 μmol TE/μmol YGD in RP assay and a TE value of 0.47 μmol EE/μmol YGD in FCA.

For in vitro chemical assays, it is worth of mentioning that in ABTS and ORAC assays, peptides with Tyr, Trp, Ala, Asp, and Cys in N‐terminal side or C‐terminal side show high radical scavenging ability (Zheng, Zhao, Dong, et al., 2016). YGD exhibited relative high abilities both in ABTS and ORAC assays mainly in virtue of the existence of Tyr in N‐terminal and Asp in C‐terminal. For Tyr, it is supposed to play an important role in high radical scavenging ability when it is at both terminals of the peptides; therefore, it can stabilize active oxygen through direct electron transfer (Zheng, Zhao, Xiao, Zhao, & Su, 2016). It is the same that peptide (LPGPILSSFPQ) isolated from feather keratin exhibited activity in ABTS (Fontoura et al., 2019), what can be found after the structure comparison with YGD is that Gly in the sequence may contribute to the capacity as well. VNP also showed ability in ORAC assay from which we deduced Val and Pro may also play an important role in the ability. However, when referring to the study of Xing's et al. (2018), Pro residue plays an essential role in ORAC value. Finally, we conclude the capacity of VNP showed in ORAC assay mainly came from Pro in the C‐terminal. In RP assay, it is reported that Trp‐peptides and His‐peptides tended to have reducing capacity (Canabady‐Rochelle et al., 2015). YGD and VNP exhibited certain abilities, from this we can conclude that Tyr, Val at N‐terminal and Asp, Pro at C‐terminal may contribute to the abilities. This may be related to peptide bonds or the charge amino and C‐terminal groups, or both, which contributed to the oxidation‐reduction mechanism (Wu, Shiau, Chen, & Chiou, 2003). While in FCA assay, His‐peptides were reported possess high chelating capacity and Trp residue was not involved in the mechanism of ferrous and iron chelation (Canabady‐Rochelle et al., 2015). YGD exhibited ferrous chelating ability; however, VNP did not exhibited capacity from which we deduced Tyr at N‐terminal and Asp at C‐terminal may contribute to the ability, and Val at N‐terminal and Pro at C‐terminal showed no ability. Tyr and Asp residue may prevent the binding of ferrozine to ferrous during the assay. Structure–activity relationship experiments need to be done to determine the specific role of Tyr, Asp, Val, and Pro they had played in these antioxidant assays.

### 3.3 Interaction assays between peptides and phenols on ABTS, DPPH, and RP assays

**Figure 4:**
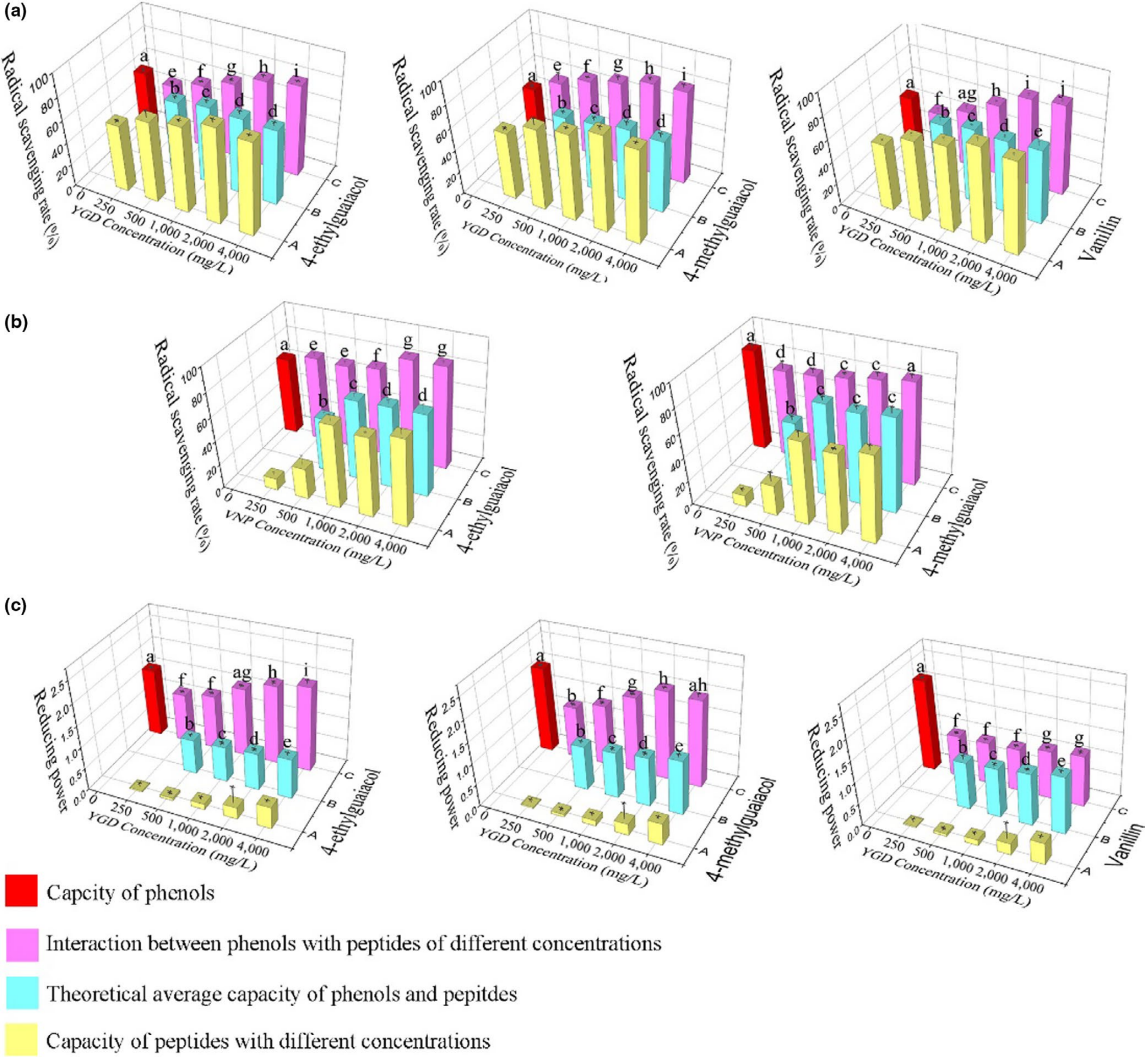
Interactions between phenols (4‐EG, 4‐MG, VA) and tripeptides (VNP and YGD) in ,2′‐azino‐bis (3‐ethylbenzothiazoline‐6‐sulfonic acid) diammonium salt (a), 1,1‐diphenyl‐2‐picrylhydrazyl (b), and reducing power (c) assays. Data are presented as the mean ± *SD*. Different letters indicate significant differences at *p* < 0.05, *n* = 3 data points

In ABTS assays, YGD, 4‐EG, 4‐MG, and VA exhibited radical scavenging activities. Therefore, the impact of YGD on 4‐EG, 4‐MG, and VA of ABTS assay was measured. Four different concentrations of YGD were mixed with 4‐EG, 4‐MG, and VA, and the radical scavenging activities were measured; the results were contrasted to the radical scavenging activity of YGD and theoretical mean of YGD with 4‐EG, 4‐MG, and VA, separately. We can see from Figure 4a, the promoted effect of YGD on 4‐EG, 4‐MG, and VA was increased with the increment of YGD concentrations. When YGD concentration arrived at 4,000 mg/L, the promoted effect almost saturated. The radical scavenging abilities of 4‐EG, 4‐MG, and VA were increased by 52.88%, 98.96%, and 70.14% to the maximum extent. The radical scavenging rate of each experimental group was higher than the theoretical average rate of YGD with 4‐EG, 4‐MG, and VA but lower than the one of YGD alone. Therefore, there was a partial promoted effect of YGD on three phenols in ABTS assay.

In DPPH assay, only VNP, 4‐EG, and 4‐MG showed radical scavenging ability. Therefore, the impact of VNP on 4‐EG and 4‐MG was studied. The results were described in Figure 4b. It was the same that the radical scavenging rate of 4‐EG was increased with the increment of VNP concentrations and saturated when VNP concentration was up to 2,000 mg/L. The radical scavenging rate of 4‐EG was increased by 32.35% to the maximum extent, and the rate of each experimental group was higher than the theoretical average rate of different concentrations VNP mixed with 4‐EG and the rate of VNP alone. Therefore, there was a completed promoted effect of VNP on 4‐EG in DPPH assay. However, the effect of VNP on 4‐MG was partial inhibition.

In RP assay, YGD and three phenols exhibited abilities. The reducing power of YGD mixed with 4‐EG, 4‐MG, and VA separately was compared with the ones of 4‐EG, 4‐MG, VA, YGD, and the theoretical average power of different concentrations YGD mixed with three phenols. From Figure 4c, there was a completed synergistic effect that YGD acted on 4‐EG with a maximum increment of reducing power by 24.27%, and it reached saturation when 4,000 mg/L YGD was mixed with 4‐EG. The ability of the mixture was higher than YGD, 4‐EG, and the theoretical average of different concentrations YGD mixed with 4‐EG. However, there was a partial inhibitory effect YGD acted on 4‐MG and VA. When 2,000 mg/L YGD was added into 4‐MG, the reducing power of the mixture was almost the same (*p* < 0.05) with the one of 4‐MG alone and the power of the mixture was saturated at the same time. When the concentrations of YGD mixed with 4‐MG were under 2,000 g/L, it was obvious that the reducing power of the peptide–phenol mixture was lower than 4‐MG alone. Compared with 4‐MG, the inhibitory effect that YGD on VA was more obvious (*p* < 0.05), the power of different concentrations YGD mixed with VA were lower than the theoretical average power of YGD‐VA mixture and only higher than that of YGD alone.

4‐EG, 4‐MG, and VA were already reported as the aroma compounds in Baijiu (Zhao et al., 2017). VNP and YGD were identified by our previous work in Jiupei (Zhang, Jiang, et al., 2018); therefore, it is possible that these two peptides will be brought into Baijiu during the distillation process. The antioxidant ability of peptides and phenols might be influenced by an interaction. This paper measured the antioxidant effect that peptides on phenols and found YGD exhibited promoted effect (4‐EG and 4‐MG in ABTS assay and 4‐EG in RP assay). VNP showed synergistic action on 4‐EG and VA in DPPH assay. Study (Liu et al., 2016) has shown that the free radical scavenging ability of phenols is related to the electron transfer and dehydrogenase ability of phenolic hydroxyl groups (PHGs). In the study, the antioxidant activities of phenols were promoted by two peptides, which may be contributed to the interaction between the active sites Tyr of YGD and Pro of VNP on the PHGs of phenols. In the process of binding, the electrostatic charge of oxygen atom in PHGs is the largest, which leads to the easy separation of O–H bond in PHGs and hydrogen extraction reaction is happened to combine with free radicals, finally the purpose of scavenging free radicals is achieved. In addition, p‐π conjugated forms can be produced between the oxygen of PHGs and benzene ring, and the negative charges of O^2−^ can be dissociated to the carbon atoms on the benzene ring and disperse the negative charge of O^2^, so PHGs are easy to release hydrogen ions to terminate the free radical chain reaction. Therefore, synergistic effect happened between two peptides and three phenols, which significantly enhance the quenching ability of free radicals (Hernández‐Jabalera et al., 2015). The above research sets as a reference before bringing functional peptides into Baijiu by optimizing distillation conditions or directly adding peptides isolated from Jiuzao (the residue after Baijiu distillation) in the future.

### 3.4 Cell viability

Val‐Asn‐Pro and YGD exhibited antioxidant activities in in vitro chemical assays; however, these assays exist some boundedness for the absence of biological relevance and the matrix environment of bioavailability (Jahandideh, Chakrabarti, Davidge, & Wu, 2016). The mechanism of action and metabolism of body system in vivo is uninvolved. Consequently, the antioxidant activities of VNP and YGD in vivo are requisite to study in biological cell system.

**Figure 5:**
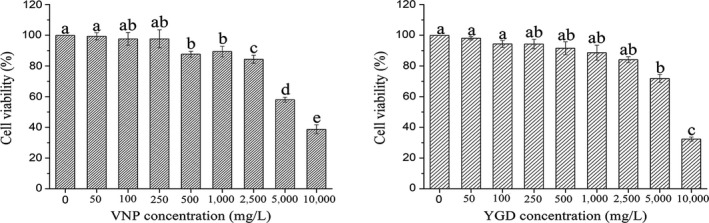
Cell viability of HepG2 cells treated with various concentrations of VNP and YGD. Data are shown as the mean ± *SD* from three independent experiments. Different letters express the significant difference at *p* < 0.05

In order to determine the appropriate peptides treatments on cells for in vivo assays, the viability of HepG2 cells was measured by CCK‐8 assay. As shown in Figure 5, after being treated with VNP and YGD for 24 hr, cell viability decreased in a concentration‐dependent manner with increasing sample concentrations. When the concentration of two peptides was 10,000 mg/L, it exhibited obvious (*p* < 0.05) cytotoxic effects on HepG2 cells. The cell viability descended evidently (*p* < 0.05) when the concentrations of VNP and YGD were up to 5,000 mg/L. Therefore, the appropriate concentration of VNP and YGD was 2,000 mg/L, which suggesting the toxicity of peptides on HepG2 cells can be negligible (cell viability >80%). In addition, another two experiments were set up using different concentrations 1,000 and 500 mg/L to further investigate the protective effects of different concentrations of peptides.

### 3.5 Protective effects of VNP and YGD on AAPH‐induced ROS generation in HepG2 cells

**Figure 6:**
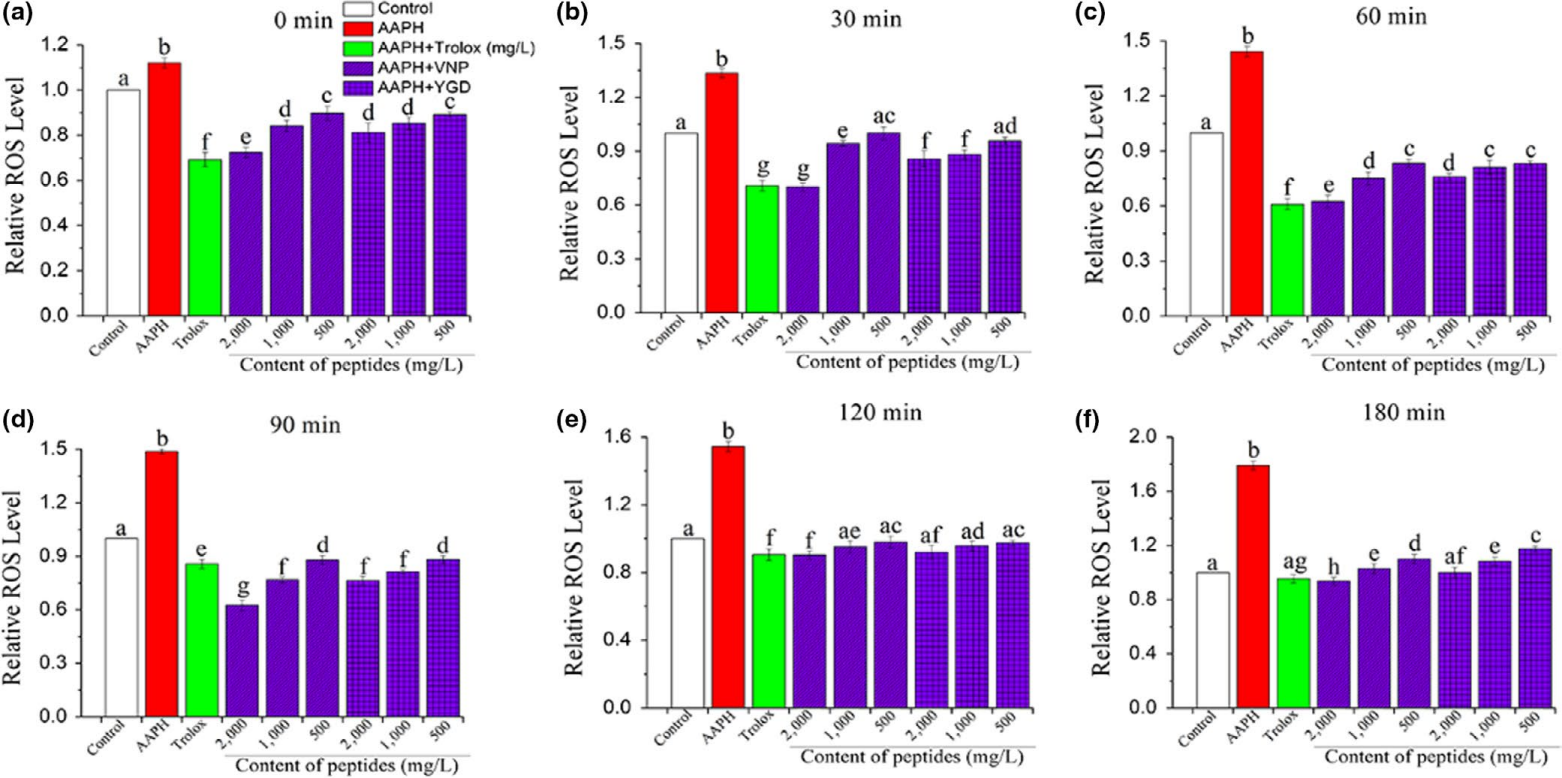
Intracellular ROS scavenging capacities against AAPH of different concentrations of VNP and YGD. The results of different incubation time (0 min [a], 30 min [b], 60 min [c], 90 min [d], 120 min [e], and 180 min [f]) were expressed as relative values versus the control group. Data were presented as the mean ± *SD* from independent assays. Different letters mean the significant difference at *p* < 0.05

In light of the observations in this study, AAPH can penetrate cell membrane and induce the production of ROS; therefore, the concentration of ROS can be used as an index to evaluate the OS in HepG2 cells. ROS are regarded as an important role material in a variety of physiological processes; thus, the protective capacities of VNP and YGD against AAPH‐induced ROS generation in HepG2 cells were measured, and the contents of two peptides and incubation time were taken into consideration. As expressed in Figure 6, the ROS content was shown as fold expression change relative to the control group, and the amount of ROS relative value increased at a stable rate within 120 min when cells were treated with AAPH. However, both Trolox group and peptides group (high, medium, and low concentrations) inhibited the raise of ROS relative value and remained lower than the control group when the incubation time was up to 120 min from the beginning. Also noted was that the ROS relative value of cells treated with VNP 2,000 mg/L exhibited almost the same level compared with Trolox group, from which we deduced VNP under this concentration expressed a quite high ROS inhibiting ability. There was nearly no significant difference (*p* < 0.05) on ROS generation in high level of YGD compared with the control group when the incubation time was up to 180 min. These findings indicated that the ROS levels decreased in a concentration‐ and time‐dependent manner with increasing concentrations of VNP and YGD. All the concentrations of two peptides significantly (*p* < 0.05) decreased ROS generation and reduced the levels of ROS to those of the control group even Trolox group cells. Similar results had been reported by Wu et al. (2017), Huo et al. (2018), who found the antioxidant peptides from Baijiu also protect HepG2 cells from oxidative damage by scavenging ROS.

### 3.6 Effect of VNP and YGD on the AAPH‐induced influence in SOD, CAT, GSH‐Px, GSH, GSSG, and MDA

In order to deeply study the effect of VNP and YGD on the AAPH‐induced HepG2 cells, the important intracellular antioxidant enzymes and no‐enzymatic OS markers in the cells were taken into consideration. SOD is an important intracellular antioxidant enzyme in organism with special physiological activity, and it is the chief substance of scavenging free radical in body (Zhang, Zhang, Wang, Xing, & Sun, 2019). SOD can catalyze the superoxide anion into less reactive oxygen and hydrogen peroxide so as to protect the cells from being damaged (Wu et al., 2017). Excess hydrogen peroxide can be decomposed by CAT and GSH‐Px, inhibited hydrogen peroxide being converted into more active substances, such as hydroxyl radicals, which accelerate the decline of cells. Therefore, activity status of the antioxidant enzymes can be used as biomarkers of the antioxidant response in the cells. In this study, the influence of different concentrations VNP and YGD on SOD, CAT, and GSH‐Px was appraised.

**Figure 7:**
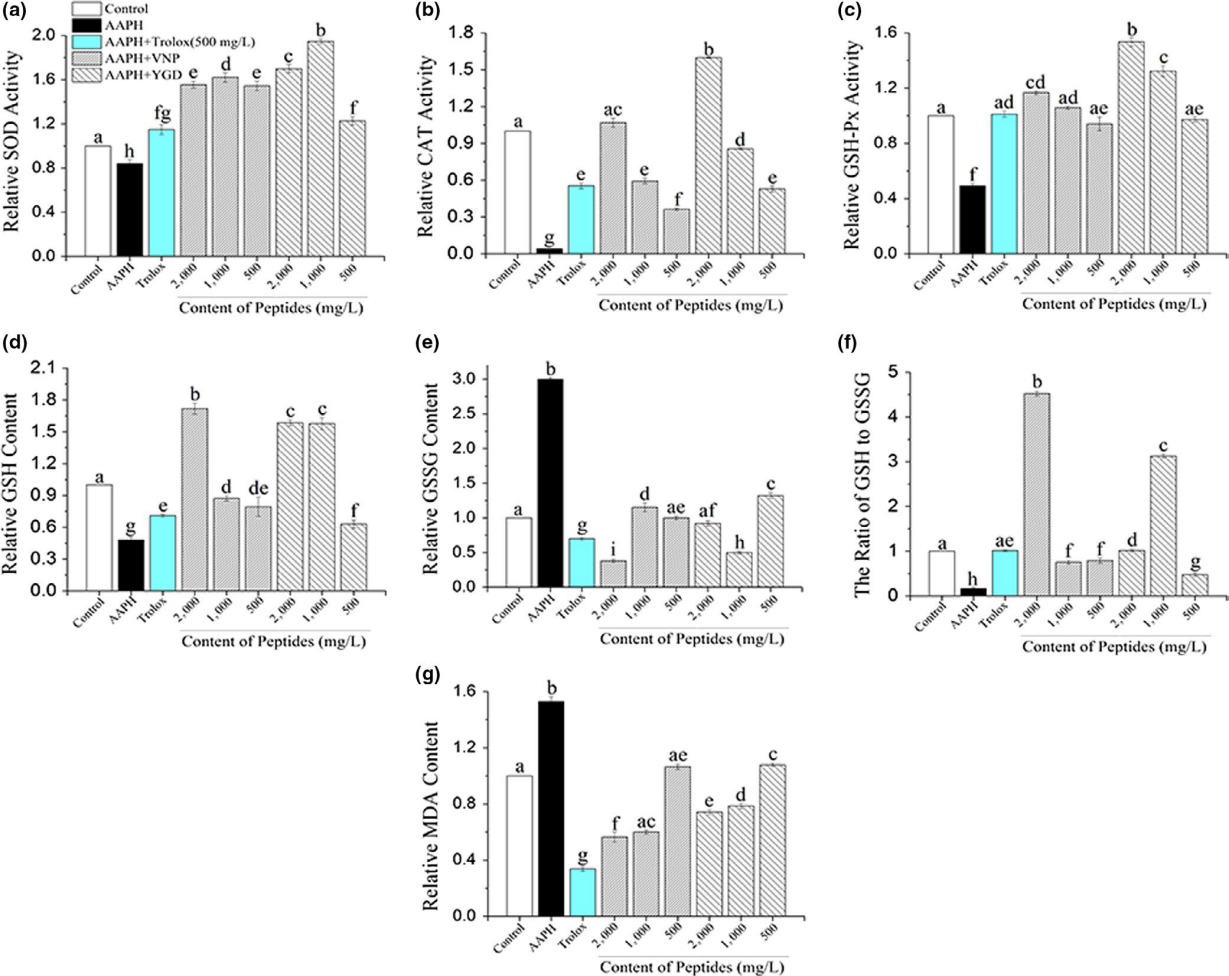
Effects of VNP and YGD on AAPH‐induced changes in SOD (a), CAT (b), GSH‐Px (c), GSH (d), GSSG (e), the ratio of GSH to GSSG (f), and MDA (g) levels in HepG2 cells. The results above are presented as relative values versus the control group. Data are expressed as the mean ± *SD* from three independent assays. Different letters mean the significant difference at *p* < 0.05

As exhibited in Figure 7a–c, the activity of SOD (17.00%), CAT (96.10%), and GSH‐Px (50.90%) was significantly decreased (*p* < 0.05) compared with the control group, which indicated that AAPH did increase the OS in HepG2 cells, similar to the study of Wu et al. (2017). However, when AAPH‐induced cells were treated with different concentrations of VNP and YGD, the activities of three enzymes increased with different degrees (*p* < 0.05). The activity of SOD was increased compared with the control group when treated with three levels (high level 2,000 mg/L, medium level 1,000 mg/L, and low level 500 mg/L) of two peptides. Among three levels, medium level of both peptides exhibited the best effect (62.21% for VNP, 94.72% for YGD) on the increment of SOD activities, followed with high level groups of two peptides (55.51% for VNP, 69.90% for YGD) and low level at last (54.52% for VNP, 22.83% for YGD). Both VNP and YGD also increased the activity of CAT and the effect increased with the raise of treated concentrations of peptides. Two peptides exhibited the best effect when the concentration was 2,000 mg/L, activity in VNP (6.91%) group returned back to normal value compared with the control group, and capacity in the YGD (59.90%) group exceed the normal value. At last for GSH‐Px assay, the activity GSH‐Px significantly (*p* < 0.05) increased with high level (16.84% for VNP and 53.55% for YGD, respectively) and medium level (5.90% for VNP and 32.37% for YGD, respectively). The effect of VNP (high level) and YGD (high and medium level) was even higher than the Trolox group. These results demonstrated that VNP and YGD exhibited preventive effects against AAPH‐induced influence in the activities of SOD, CAT, and GSH‐Px. Overall, peptides’ effect on the activities of these antioxidant enzymes increased with the pretreated concentrations, except in SOD assays, when medium concentration made better effect compared with high concentration, from which we deduced that the effect was saturated when the concentrations of the peptides were around 1,000 mg/L.

It is important to note that activities of SOD, CAT, and GSH‐Px in two peptides high level groups were significantly (*p* < 0.05) higher than Trolox group. The antioxidant enzyme activity in medium level treatments also exhibited almost the same effect compared with the Trolox group. Liang, Zhang, and Lin (2017) also observed that peptide QDHCH identified from pine nut increased the activity level of SOD, CAT, and GSH‐Px by 91.92, 7.98, and 18.50 U/mg prot. This is in accord with the study above that peptides (VNP and YGD) can also effectively enhance the activities of the antioxidant defense systems of HepG2 cell model by increasing the activities of SOD, CAT, and GSH‐Px.

Glutathione exists widely in animals and plants and plays an important role in organisms, acting as the main nonenzymatic antioxidant defense in the cells. Under the catalysis of GSH‐Px, GSH can reduce H_2_O_2_ to H_2_O, with a change to oxidized form GSSG. GSSG then can be reduced back to GSH by accepting H^+^, so that the free radical scavenging reaction in the body can be sustained (Claeson, Gouveia‐Figueira, Stenlund, & Johansson, 2019; Lee, Kim, & Hwang, 2014). Under normal condition, the increase in GSH concentration can be deemed the cell protection against potential oxidative damage. Thus, the contents of both GSH and GSSG were measured in the study. As it shown in Figure 7d and e, a rapid decrease on cytoplasmic GSH content and increment on GSSG content in AAPH group indicated oxidation happen in the cells. However, compared with the AAPH group, the contents of GSH in VNP and YGD groups were significantly increased (*p* < 0.05; 5.6 times, 5.9 times, and 5.8 times for VNP; 5.4 times, 7.4 times, and 5.5 times for YGD, respectively), and the contents of GSSG in VNP and YGD groups were significantly decreased (*p* < 0.05; 7.9 times, 2.6 times, and 3 times for VNP; 3.2 times, 6 times and, 2.2 times for YGD, respectively). GSSG contents in high level of VNP (0.105 ± 0.004 μmol/L) and medium level of YDG (0.13 ± 0.01 μmol/L) also exhibited effects of decreasing GSSG. These results illustrated that VNP and YGD could promote the nonenzymatic antioxidant system in the cells with appropriate concentrations.

To deeply study the influence of VNP and YGD on the level of GSH and GSSG in HepG2 cells, the ratio of GSH to GSSG was also measured. As shown in Figure 7f, the lowest ratio of GSH to GSSG was in the AAPH group (0.15 ± 0.01). A significant difference (*p* < 0.05) was even found in low level peptide group and AAPH group; this indicated that VNP and YGD exhibited effect of converting GSH to GSSG. High level in VNP showed strongest effect (4.50 ± 0.03), and there is no significant difference between medium and low levels in VNP. While for YGD, the most effect group was medium level (3.13 ± 0.02). From the result above, we can conclude that appropriate concentrations of VNP (2,000 mg/L) and YGD (1,000 mg/L) exhibited strong effect in preventing GSH decreasing and GSSG increasing, which helped the cells inhibit oxidative damage. The similar result was found in other studies (Du, Esfandi, Willmore, & Tsopmo, 2016; Wu et al., 2018) that the increased generation of GSH and the decreased level of GSSG could be promoted by peptides.

Malondialdehyde is one of the most important products of membrane lipid peroxidation, and its production can also aggravate membrane damage. Therefore, MDA content is a common index in cell senescence and resistance physiology; the degree of membrane lipid peroxidation can be measured according to the content of MDA so as to evaluate the damage to membrane system and resistance of cells indirectly (Adnan et al., 2019; Mahendra et al., 2018). As exhibited in Figure 7g, in the AAPH group there evoked a significant increment (1.5 times) of MDA content when compared with the control group. However, when treated with high and medium levels of VNP or YGD for 3 hr, the content of MDA decreased (39.99%–43.52% for VNP, 21.46%–25.74% for YGD). This result revealed that the level of AAPH‐induced lipid peroxidation in peptides groups (high and medium level) was inhibited with a certain degree. Similar result was found in pine nut peptide QDHCH (Liang et al., 2017), which significantly restrained the increase in MDA content.

When pretreating HepG2 cells with oxidative damage caused by AAPH, the ROS relative value in the cells of peptides (VNP and YGD) groups exhibited a downward trend compared with the AAPH group. This trend was dependent on the concentrations and incubation time of the sample. The ROS relative value in the cells of peptides group was lower than the control group during a period of time under three levels of concentrations, which indicated that VNP and YGD had effect on clearing the damaged ROS directly. VNP and YGD can increase the intracellular antioxidant enzymatic system (SOD, CAT, and GSH‐Px) activity and moderate the contents of GSH, and GSSG also decreases the content of MDA, while the effect of peptides on the enzymatic and nonenzymatic systems is not always a positive correlation with concentrations. When comparing VNP with YGD, YGD exhibited stronger effect (*p* < 0.05) on the intracellular antioxidant enzymatic systems; we deduced that Tyr in N‐terminal and Asp in C‐terminal may contribute to the effect more than Val and Pro. However, VNP showed stronger effect on the increase in GSH content and decrease in GSSG also MDA content in the cells; this may indicate that Val in N‐terminal and Pro in C‐terminal affects the formation than Tyr and Asp.

## 4 CONCLUSION

To sum up, the antioxidant capacities of two tripeptides VNP and YGD identified in Jiupei were investigated by in vitro chemical assays and AAPH‐induced HepG2 cell model; the interaction between peptides (VNP and YGD) and functional phenols (4‐EG, 4‐MG, and VA) were also evaluated by in vitro chemical assays. Two tripeptides exhibited strong antioxidant activities in ORAC assay. In the AAPH‐induced HepG2 cell model, VNP and YGD exert antioxidant effect on nonenzymatic antioxidant system (GSH, GSSG, and MDA) and enzymatic antioxidant system (SOD, CAT, and GSH‐Px). There was a partial promoted effect of VNP and YGD on the antioxidant effect of 4‐MG, 4‐EG, and VA. The results in this study provide a reference to study a series of interaction among different functional substances in Baijiu and lay the foundation for better increasing the health protective properties (e.g., antioxidant peptides) of Baijiu from distillation process.

## CONFLICT OF INTEREST

No potential conflict of interest was reported by the authors.

## ETHICAL APPROVAL

The protocols and procedures were ethically reviewed and approved by Beijing Technology and Business University. There was no human testing or animal testing in the study; ethics approval and consent to participate are not applicable to this manuscript.
